# *De Novo* Origin of *VCY2* from Autosome to Y-Transposed Amplicon

**DOI:** 10.1371/journal.pone.0119651

**Published:** 2015-03-23

**Authors:** Peng-Rong Cao, Lei Wang, Yu-Chao Jiang, Yin-Sha Yi, Fang Qu, Tao-Cheng Liu, Yuan Lv

**Affiliations:** 1 Department of Epidemiology, Medical College of Hunan Normal University, Changsha, China; 2 The State Key Laboratory of Genetic Engineering and MOE Key Laboratory of Contemporary Anthropology School of Life Sciences, Fudan University, Shanghai, China; University of Münster, GERMANY

## Abstract

The formation of new genes is a primary driving force of evolution in all organisms. The *de novo* evolution of new genes from non-protein-coding genomic regions is emerging as an important additional mechanism for novel gene creation. Y chromosomes underlie sex determination in mammals and contain genes that are required for male-specific functions. In this study, a search was undertaken for Y chromosome *de novo* genes derived from non-protein-coding sequences. The Y chromosome orphan gene variable charge, Y-linked (*VCY*)*2*, is an autosome-derived gene that has sequence similarity to large autosomal fragments but lacks an autosomal protein-coding homolog. *VCY2* locates in the amplicon containing long DNA fragments that were transposed from autosomes to the Y chromosome before the ape-monkey split. We confirmed that *VCY2*cannot be encoded by autosomes due to the presence of multiple disablers that disrupt the open reading frame, such as the absence of start or stop codons and the presence of premature stop codons. Similar observations have been made for homologs in the autosomes of the chimpanzee, gorilla, rhesus macaque, baboon and out-group marmoset, which suggests that there was a non-protein-coding ancestral *VCY2* that was common to apes and monkeys that predated the transposition event. Furthermore, while protein-coding orthologs are absent, a putative non-protein-coding *VCY2* with conserved disablers was identified in the rhesus macaque Y chromosome male-specific region. This finding implies that *VCY2* might have not acquired its protein-coding ability before the ape-monkey split. *VCY2* encodes a testis-specific expressed protein and is involved in the pathologic process of male infertility, and the acquisition of this gene might improve male fertility. This is the first evidence that *de novo* genes can be generated from transposed autosomal non-protein-coding segments, and this evidence provides novel insights into the evolutionary history of the Y chromosome.

## Introduction

The emergence of new genes with novel functions is important for adaptive evolutionary innovation [1]. Using preexisting genes as raw materials, new genes can arise in various manners, such as exon shuffling, gene duplication, retroposition, lateral gene transfer, gene fusion, and fission [2]. It has recently become apparent that new genes can also arise *de novo* from non-protein-coding DNA; examples have been reported in fruit flies (*Drosophila*) [3–8], ants and other insects [9], budding yeast (*Saccharomyces cerevisiae*) [10–12], rice (*Oryza sativa*) [13], protozoal parasite *Plasmodium vivax* [14], mice (*Mus musculus*) [15], primates, and humans. For example, 15 *de novo* protein-coding genes have identified in the ancestral primate genome using comparative genomic analyses [16], while other studies have found three putative protein-coding genes in humans and 11 novel genes in the murine genome [17,18], and *de novo* protein-coding genes involved in brain function [19] and with tumor-specific expression have been identified [20]. A comparative analysis of primate genomes found 60 putative human-specific genes [21]. Hominoid-specific novel protein-coding genes originating from long non-protein-coding RNAs [22] have also been identified. The possible *de novo* protein-coding genes on chromosome Y (Chr-Y) aroused our interest because Chr-Y underlies sex determination in mammals and contains genes that are specialized for the male sex and reproduction [23]. However, there have been no such reports of *de novo* protein-coding genes on the Chr-Y, which harbors genes for male-specific functions.

The mammalian sex chromosomes evolved from an ancestral, identical homologous pair (proto-X/Y). After acquiring the male sex-determining gene sex-determining region of the Y-chromosome (SRY), meiotic recombination was suppressed through sequential inversions of segments of the mammalian chromosome X (Chr-X) and Chr-Y [24,25]. This process halted recombination between groups of contiguous X chromosomal genes and their Y chromosomal homologs. This event ensured that the male-specific gene on the Chr-Y were restricted to male. This event is referred as a stratum, and it has been estimated that there were four X chromosomal strata in the evolutionary history of primates and other mammals [26,27]. Only a small portion of human Chr-Y can still recombine with Chr-X. The pseudoautosomal (PAR) region in the short arm of Chr-Y is the only remaining locus for recombination and contains over 29 genes with identical copies on the Chr-X [28–30]. The non-recombining male-specific region of Chr-Y (*MSY*) comprises approximately 95% of the total chromosome length and contains 78 protein-coding genes that encode 27 distinct proteins [31]. *MSY* euchromatic sequences can be classified as X-transposed, X-degenerate, and ampliconic regions. The X-transposed region in humans contains only two genes that are thought to have been generated by a recent transposition from Chr-X and exhibit 99% identity to Xq21 [32]. The X-degenerate region is considered to be a remnant of the degeneration of the ancestral mammalian proto-Y [33]. A total of 16 X-degenerate Chr-Y genes with X-linked homologs are found in humans and gorillas, and 12 are found in chimpanzees [34,35]. Some ampliconic genes in Chr-Y were generated through amplifications of X-degenerate genes, such as the RNA binding motif protein Y-linked 1 (*RBMY1A1*) [36,37]. Other male-benefit genes might have arisen through autosomal transposition and amplification, for example, deleted in the azoospermia protein (*DAZ*) gene cluster [38,39], or by retroposition like testis-specific chromodomain protein Y-linked 1 (*CDY*) [40,41]. Those genes could improve the male reproductive ability and lost of these genes might cause the reduction of the reproductive ability or male infertility. Because Chr-X and autosomes both shaped the gene content of Chr-Y (through the degeneration of the proto-X/Y gene pair and the retroposition and transposition of autosomal copies, respectively), we searched for evidence of *de novo* protein-coding genes in Chr-X and autosomes and confirmed the *de novo* origin of Chr-Y genes.

Previously described analysis strategies [15–19] were used in the present study to search for possible *de novo* protein-coding genes on human Chr-Y, but they were modified according to the unique evolutionary history of Chr-Y. The search encompassed not only human-specific genes with non-genic orthologous sequences in sister primate lineages (i.e., humans Chr-Y [42], chimpanzees [35] and rhesus macaques Chr-Y male-specific region [43]) but also considered the origins of Chr-Y genomic sequences (i.e., autosomes and Chr-X). In this study, we searched for Chr-Y *de novo* genes from non-protein-coding sequences acquired through retroposition or transposition from autosomes or from the X-degenerate region. We provide evidence that the autosomal-transposed orphan gene *VCY2* might have arisen *de novo* from non-protein-coding regions.

## Materials and Methods

### Data collection

The primate genome sequences from the National Center for Biotechnology Information (http://www.ncbi.nlm.nih.gov/) [44] and Ensembl databases (www.Ensembl.org/) [45] were extracted and analyzed. The genomic assemblies version referred to in this article and the detailed the genomic sequence information we used in this article are listed in S1 Table. The bacterial artificial chromosome (BAC) clones of rhesus macaque Y chromosome male-specific region referred to in this article and the sequence information are listed in S2 Table. Rhesus macaque Chr-Y testis cDNA sequences with accession numbers FJ527009-FJ527028 and FJ648737-FJ648739 in GenBank (www.ncbi.nlm.nih.gov) [44] were used to verify the rhesus macaque *VCY2* gene expression. The expressed sequence tag (EST) from human [46] and cDNA clones from chimpanzee (chimpanzee testis library Koos) [47] in the GenBank (www.ncbi.nlm.nih.gov/genbank) were used for the *VCY2* and *BEYLA* expression evidence. The marmoset testis EST clone set materials were provided by the RIKEN BRC through the National Bio-Resource Project of the MEXT, Japan[48]. The mRNA transcription evidence for *VCY2* was collected from the transcriptome shotgun assembly database [24]. The expression profile of the non-protein-coding *BEYLA* (NONCODE GENE ID:NONHSAG050144) was exacted from the non-coding RNA database NONCODE (http://www.bioinfo.org/noncode/) [49].

### Sequence analysis

The sequences were assembled using MEGA5 (Gene Codes, Ann Arbor, MI, USA) and aligned with ClustalW [50]. Phylogenetic analyses were performed using the maximum likelihood (ML) method. Phylogenetic trees were constructed with the Kimura two-parameter model. The gamma-distributed with invariant sites (G+I) Tamura-Nei model [51–54] was used for the maximum likelihood analyses. Bootstrap test results with 500 replicates are shown next to the branches of the trees. The evolutionary analyses were performed with MEGA6. The dot-plot comparisons between pairs of sequences were drawn with DNAMAN program (Lynnon Biosoft, Quebec, Canada) with default parameters.

### Primate Chr-Y orphan genes

Human, chimpanzee and rhesus macaque Chr-Y protein-coding genes were retrieved from the National Center for Biotechnology Information (http://www.ncbi.nlm.nih.gov/) and Ensembl databases (www.Ensembl.org/). The orthologs from the non-primate species were identified through a Basic Local Alignment Search Tool Protein-Protein (BLASTP) [55–58] search against a non-primate non-redundant (nr) protein sequences database using an E-value threshold of 10^-4^ and a sequence identity greater than 35%. The genes without any possible homologs in non-primate species were considered to be primate-specific Chr-Y orphan genes.

### Candidate *de novo*-originated primate Chr-Y genes

The Chr-Y orphan genes that were restricted to the primate phylogenetic lineage were examined to evaluate whether they could have arisen *de novo*. A second round of BLAST was performed to search against the primate non-redundant (nr) protein sequences database using an E-value threshold of 10^-4^ and a sequence identity greater than 35%. The corresponding genomic location of each hit was analyzed. If the hit was located on an autosome or Chr-X, the gene was excluded from further analyses. The genomic sequences of the remaining genes were verified using BLAST and the BLAST-like alignment tool (BLAT) algorithm against the primate genomes from the National Center for Biotechnology Information (http://www.ncbi.nlm.nih.gov/) and the UCSC Genome Browser (http://genome.ucsc.edu/) databases [59] to identify the sequences with high similarities. The genomic fragments (lengths over 4 kb and identities over 80%) were used for subsequent analyses. The protein-coding abilities of these regions were further investigated.

### Identification of the homologs and synteny analysis

Homologs are identified through both sequences similarity comparisons and syntenic analyses. A BLAST was performed to search against the primate non-redundant (nr) protein sequences database using an E-value threshold of 10^-4^ and a sequence identity greater than 35%. The corresponding genomic location of each hit was analyzed. The analysis of relative gene-order conservation between species was carried. Synteny analyses were performed on 9 representative primate species that included humans (*Homo sapiens*), chimpanzees (*Pan troglodytes*), bonobos (*Pan paniscus*), gorillas (*Gorilla gorilla*), Norther white-cheeked gibbons (*Nomascus leucogenys*), olive baboons (*Papio anubis*), rhesus macaques (*Macaca mulatta*), sabaeus monkeys (*Chlorocebus sabaeus*) and common marmosets (*Callithrix jacchus*). Synteny maps of the conserved genomic regions in the primates were performed using the Genomicus v67.01 web site [60].

## Results

### Hypotheses for the origin of Chr-Y *de novo* protein-coding genes

**Fig 1:**
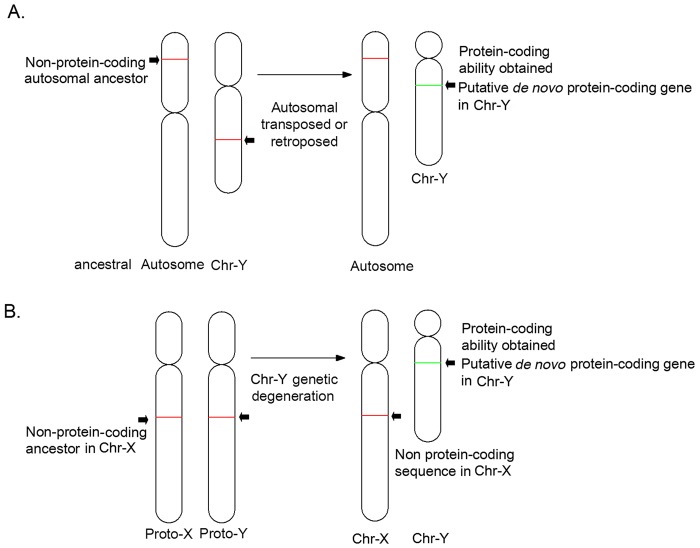
Model of the origin of *de novo* protein-coding genes on Chr-Y. A putative *de novo* protein-coding gene (green) acquired the protein-coding ability through (A) the transposition or retroposition of non-protein-coding autosomal segments or (B) X-degenerate sequences from an ancestral proto-sex chromosome, while non-protein-coding homologs remained so on autosomes or Chr-X (red).

We hypothesized that if a protein-coding gene had arisen *de novo* from non-protein-coding sequences acquired through retroposition or transposition from autosomes, then non-protein-coding autosomal copies would persist (Fig. 1A). Similarly, if a protein-coding gene in the X-degenerate region had been generated *de novo* from a proto-X/Y sequence, then non-protein-coding copies would be found on Chr-X (Fig. 1B).

### Candidate *de novo* protein-coding genes on Chr-Y

**Fig 2:**
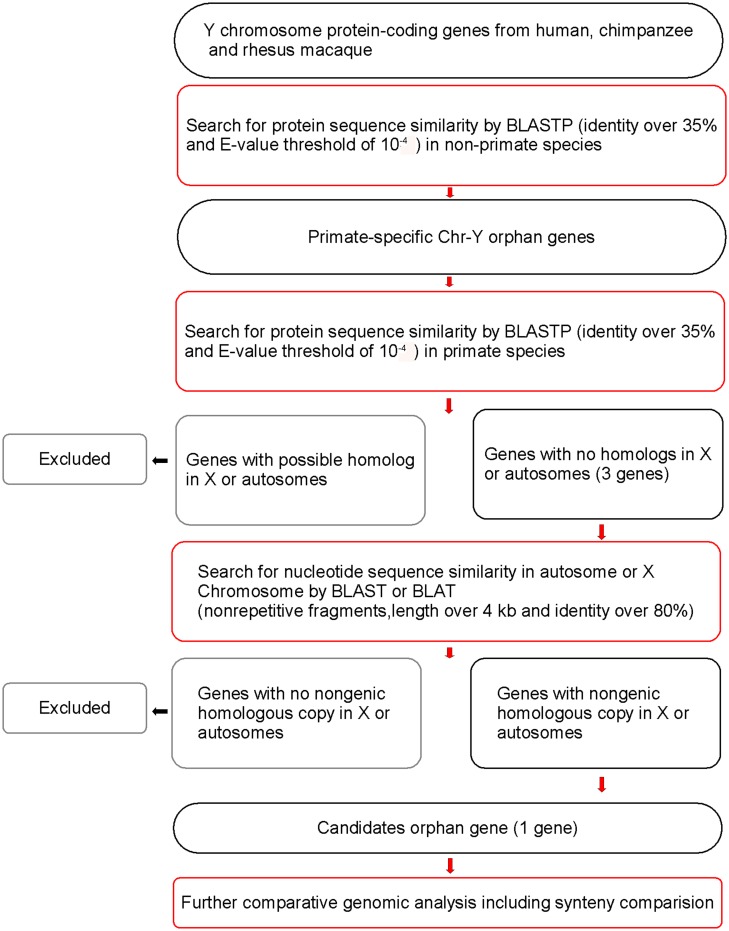
The schematic of analysis pipeline. The orthologs of primate Chr-Y genes from the non-primate species were identified through a Basic Local Alignment Search Tool Protein-Protein (BLASTP) search against a non-primate non-redundant (nr) protein sequences database using an Expect (E)-value threshold of 10^-4^ and a sequence identity greater than 35%. The genes without any possible homologs in non-primate species were considered to be primate-specific Chr-Y orphan genes. These candidates were subsequently scrutinized for any evidence of a protein-coding homolog in Chr-X or autosomes. Those genes would be excluded if there was a protein-coding gene in primate autosomes or Chr-X. The synteny comparison and sequence alignments of the candidate genes and its non-protein-coding homologs on Chr-X or autosomes would be used subsequently as the supporting information for *de novo* origin.

First, human, chimpanzee and rhesus macaque Chr-Y orphan genes were collected. Eight human orphan protein-coding genes were identified on Chr-Y through a BLAST search against the non-primate non-redundant protein sequences database as shown in Table 1. According to the deduced polypeptide sequence similarities, these genes were furth er classified into the following four distinct orphan gene families: *VCY* (variable charge, Y-linked), *VCY2* (approved gene symbol *BPY2*); protein tyrosine phosphatase, non-receptor type 3-like, Y-linked (*PRY*), and proline-rich and Y-linked (*PRORY*). The genes from each of the families, including pseudogenes, are listed in Table 1. According to our hypotheses, the potential *de novo* candidates among these orphan genes should only have sequences with high similarities with human or other primate Chr-X or autosomes and have no protein-coding homologs (including possible direct paralogs or gametologs). We then used the genomic sequences of orphan genes from each of the families, i.e., *VCY*, *VCY2*, *PRY* and *PRORY*, to search for the non-protein-coding sequences with high similarities via BLAT against the primate genomic databases. Among these four gene families, only *VCY2* exhibited high sequence similarities with autosomal fragments. The genomic locations for each fragment longer than 4 kilobase pair are listed in S3 Table. A schematic of the analysis pipeline for the candidate *de novo* protein-coding genes is shown in Fig. 2.

**Table 1:** Y-chromosomal orphan gene families that restrict to the primate lineage.

Gene family names and family members on human genome	X-linked gametologs	Autosomal gametologs or paralogs	Species with the possible Y orthologs (Cortez et al. *Nature*. 2014 and Bhowmick et al. *Genome Res*. 2007)
*VCY2* (A/B/C/D^Ψ^)	None	None	Humans, chimpanzees
			gorillas, orangutans
*PRORY*	None	None	Humans, chimpanzees
*PRY* (1/2,3/4^Ψ^)	None	None	Humans, Old World monkeys
*VCY* (1/2)	*VCX*,2,3A/B	None	Humans, chimpanzees

Y-chromosomal orphan gene families including *PROY* (proline rich, Y-linked), *PRY* (PTPN13-like, Y-linked) and *VCY* (variable charged Y-linked) 2 were restrict to the primate lineage.

^Ψ^Pseudogenes;

### Y-chromosomal *VCY2* arose via transposition of autosomal fragments

**Fig 3:**
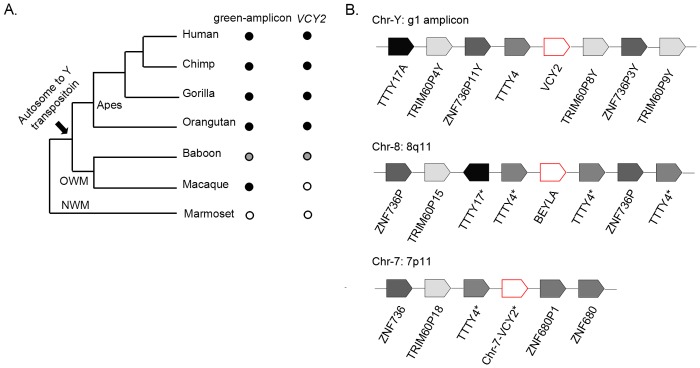
Y-chromosomal *VCY2* arose via transposition of the autosomal fragments. (A) The presence (black dots), absence (white dots) of Chr-Y green-amplicon homologous sequences and *VCY2* homologs in representative primate species. The assumed date for the amplicon transposition was indicated by the arrow at the species tree of the primates. Status unknown (gray dots). (B) The synteny analysis of the green-amplicon from human Chr-Y and autosomal fragments from 8q11 and 7q11. We marked those autosomal sequences that have high sequence similarities with Chr-Y *TTTY17* and *TTTY4* but not annotated as genes. NWM, New World monkey; OWM, Old World monkey; *P*, pseudogene; TRIM, tripartite motif containing; *TTTY*, testis-specific transcript, Y-linked; *ZNF*, zinc finger protein.

Among these four Chr-Y orphan gene families, only *VCY2* exhibited sequences with high similarities to those of the autosomes and lacked of protein-coding homologs. Three *VCY2* protein-coding paralogs (*VCY2A*, *2B*, and *2C*) and one inferred pseudogene (*BPY2DP*) were present in human Chr-Y. Two paralogs of *VCY2* in the chimpanzee were also identified (Table 2). The origin of *VCY2* has been discussed in previous studies. According to the inventories of the Chr-Y genes, *VCY2* belongs to the added gene group that entered the Chr-Y in a lineage-specific manner during evolution in contrast to the ancestral genes that are shared with other lineages. The *VCY2* gene families contained three protein-coding copies (Table 2) and mapped to the long duplicated and inverted genomic fragments of Chr-Y that can be referred to as amplicons. Some of these amplicons exhibit high sequence similarities with the autosomes and are thought to have arisen from autosomal fragment transposition and duplication. Previous studies have revealed that the ancestral amplicons arrived in Chr-Y at different times during the primate evolution and that they can be further classified into blue, light-blue, red, green and yellow amplicons [38]. *VCY2* was located in the green-amplicons. The ancestral green-amplicon was inverted and duplicated several times during the evolutionary history of Chr-Y to generate the green-1, green-2 and green-3 amplicons (g1, g2 and g3); thus, paralogs of *VCY*2 (*VCY2A*, *2B* and *2C*) were found in the Chr-Y and were each located in different amplicons. The origin of the pseudogene *BPY2DP* is unknown. The green-amplicon homologous sequences were found in the Chr-Ys of all of the great apes and Old World monkeys but were not found in the New World monkeys (out-group) (Fig. 3A). It is believed that the ancestral green-amplicon arrived in Chr-Y through autosomal duplication and transposition in the common ancestor of apes and Old World monkeys after the divergence of the New World monkeys approximately 35 million years ago (mya) [38].

**Table 2:** *VCY2* gene families in human and chimpanzee genomes.

Gene names^a^	Ensembl IDs	Chromosomal locations
*Hosa-VCY2*	ENSG00000183753	Y:25119966–25151612:1
*Hosa-VCY2B*	ENSG00000183795	Y:26753707–26785354:1
*Hosa-VCY2C*	ENSG00000185894	Y:27177048–27208695:-1
*Hosa-BPY2DP* ^Ψ^	ENSG00000229745	Y:7926089–7948999:-1
*Patr-VCY2*	ENSPTRG00000028847	Y:3959516–3965432:-1
*Patr-VCY2B*	ENSPTRG00000028812	Y:12587744–12593658:1

*VCY2* from human (GRCh38) and chimpanzee (CHIMP2.1.4) genomes were extracted and their Ensembl IDs and chromsomal location were shown. ‘1’ or ‘-1’ indicate the forward and reverse strand on the chromosome respectively. Human *BPY2DP* (basic charge, Y-linked, 2D) is a pseudogene of *VCY2*.

^a^All protein-coding paralogs encode a 106-a.a. Protein.

^Ψ^Pseudogenes.

Hosa, *Homo sapiens*; Patr, *Pan troglodytes*.

### The green amplicon-homologous human autosomal fragments

To clarify the origin of *VCY2*, we sought to examine the genomic locus before transposition. Previously, Stuppia et al. reported that chromosome 8q11.2 exhibits homology to the green-amplicon that contains the *VCY2*, *TTTY4*, and *TTTY17* genes. The *BEYLA* gene, also known as long intergenic non-protein-coding RNA 293 (LINC00293) was identified and contained autosomal fragments that exhibited high sequence similarities with *VCY2* [61]. Moreover, a shorter fragment in Chr-7 also exhibited sequence similarities with *VCY2* (S3 Table). However, unlike Chr-8 *BEYLA*, this sequences from Chr-7 had not been annotated as a gene; therefore, we named it *Chr-7-BEYLA*. To confirm their homologous relationship, we performed synteny comparison analysis centered on *VCY2*, *BEYLA* and *Chr-7-BEYLA*. The order and orientation of each gene, including these pseudogenes and the non-protein-coding RNA genes, in the whole green amplicon are shown in Fig. 3B. The *VCY2*, *BEYLA* and *Chr-7-BEYLA* sequences were positioned in genomic regions that contained common loci, including *ZNF736* and *TRIM60* (pseudogene), and thus exhibited a conserved synteny. Chr-Y genes *TTTY4* and *TTTY17* in the green-amplicon also exhibited high similarities with sequences in Chr-8 and *BEYLA* in Chr-7. We also compared 200 bp flanking sequence of *VCY2* gene with outgroup flanking sequences from Chr-8 (*BEYLA*) and Chr-7 (S1 Fig.). These results support the homologous relationship between these two autosomal fragments and the green amplicon. Subsequently, we focused on the sequence differences between *VCY2* and its homologous region in these autosomal fragments.

### Expression evidence for *VCY2* and *BEYLA*

Before we began the subsequent analysis, we examined the expression evidence for *VCY2*. The human *VCY2* gene encodes a functional protein, and its expression has previously been detected in testicular biopsy specimens [62]. VCY2 protein binding partners, such as microtubule-associated protein 1S (MAP1S) [63] and ubiquitin protein ligase E3A (UBE3A) [64] have also been identified. Two EST clones from human testis tissue are thought to belong to *VCY2* (Table 3) [46]. Regarding *BEYLA*, we found over 30 EST clone sequences that matched the human *BEYLA*, and the majority of these sequences were also from human testis tissues. Ten of these sequences are also listed in Table 3. We also found an EST clone from human testis tissues that matched Chr-7 *BEYLA*; we thus believe an unidentified testis-expressed non-protein-coding gene might exist (Table 3). The expression profile of *BEYLA* was also examined against the non-coding RNA database NONCODE, and its expression in testis tissue was much higher than its expression in other tissues (S4 Table).

**Table 3:** Expression evidence for human *VCY2* and *BEYLA* genes.

Gene names	Full names	EST evidence (origin tissues)	Other evidence for the expressions
*Hosa-VCY2*	Variable charge, Y-linked, 2	DB047517.1 and DB341225.1 (testis)	Western blot analysis and immunostaining of the testicular tissue (Tse et al. *Biol Reprod*. 2003)
*Hosa-BEYLA*	Long intergenic non-protein coding RNA 293	A105405.1, A1026871.1, A1214380.1, CD557729.1, AA905547.1 and AA412219.1 (all from testis)	Real time-PCR analysis on RNA from testis, leukocytes, prostate, kidney, placenta, ovary, brain, heart, and muscle (Stuppia et al. *Genomics*, 2005)
*Hosa-Chr-7-BEYLA*	Not annotated as a gene	DB340221 (testis)	None

The EST (expressed sequence tag) and other evidence for human *VCY2* and *BEYLA* expression was shown. The accession number for each EST clone was listed.

Hosa (*Homo sapiens*).

The RNA expression of *VCY2* was observed in chimpanzees, gorillas and orangutans but not in rhesus macaques or marmosets as shown in Table 4. The evidence of VCY2 expression at protein level was only found in human by now. We did not find any EST clone from non-human primates that matched *BEYLA*. To confirm this finding, we used the *Hosa-VCY2* cDNA and genomic sequence in a BLAST search against the rhesus macaque testis cDNA and confirmed that the *VCY2* protein-coding homolog was absent in the rhesus macaque. We did not find sequences from the marmoset testis EST clone library or the transcriptome shotgun assembly database that matched *VCY2*. Previous studies have also reported that *VCY2* is absent as a protein-coding gene in the MSY genes of the rhesus macaque, an Old World monkey [43]. While the green-amplicon homologous sequences were found in the Chr-Y of the Old World monkeys, the *VCY2* ortholog from the rhesus macaque was absent. This finding raised a question about the protein-coding ability of *VCY2* in the evolutionary history after transposition from autosomes.

**Table 4:** Expression evidence for non-human primate *VCY2* homologous genes.

Species	GI numbers	Expression levels	Sources of the data
chimpanzees	62287768	RNA (partial cds)	Reverse transcript and testis cDNA sequencing data
gorillas	584599275	RNA	Transcriptome Shotgun Assembly database (T: 4854058)
bornean orangutans	584599417	RNA	Transcriptome Shotgun Assembly database (T: 1503658)
rhesus macaques	absent	absent	Testis cDNA clones (T: 5021608)
common marmosets	absent	absent	Testis and other tissue EST clone library and transcriptome shotgun assembly database (T: 290426)

The EST (expressed sequence tag) and other evidence for non-human primate *VCY2* and *BEYLA* expression was shown. The GI (genInfo identifier) number was listed. To show the depth for each dataset, total number of the raw contigs or EST clones from the dataset (T) was shown.

### Non-protein-coding autosomal *VCY2* from humans and primates

**Fig 4:**
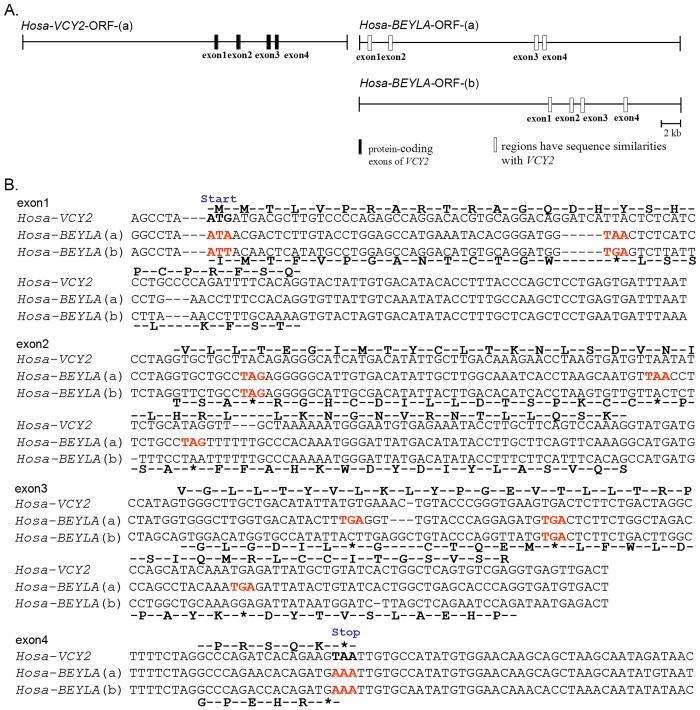
Non-protein-coding autosomal sequences homologous to *VCY2* (*BEYLA*) from 8q11 in humans. (A) The *Hosa-BELYA* sequence homologous to *Hosa-VCY2* was extracted and protein-coding exons were joined based on the *Hosa-VCY2* ORF to obtain *Hosa-BEYLA* ORF(a) and (b). (B) Map of the positions of *Hosa-VCY2* protein-coding exons (black bars) and putative exons from autosomal homologs (white bars); 1 cm in the diagram = 2 kb in the genomic sequence. Artificial translations of *Hosa-BEYLA* ORF(a) and (b) for each exon are shown below each row of the alignment and compared to the deduced protein sequence of the *Hosa-VCY2* ORF above the row. Start and stop codons are indicated. The asterisk (*) denotes the premature stop codon in the translated sequence. Disablers are indicated by red. Hosa, *Homo sapiens*.

To further investigate the origin of *VCY2*, the genomic sequences of *Homo sapiens* (*Hosa*)-*VCY2* and the sequence from the autosomal *Hosa*-BEYLA were aligned to identify the critical differences that might affect protein coding. The coding sequence (CDS) and the autosomal sequences with high similarities were used for further analysis. The putative ORF for *Hosa-VCY2* was reconstructed and joined in the order and orientation of the corresponding sequences with high similarity from the *Hosa-BEYLA* protein-coding exons. Two complete sets of putative ORFs, ORF(a) and (b), with four putative protein-coding exons were identified from *Hosa-BEYLA* (Fig. 4A).

The divergence between *Hosa-VCY2* and the autosomal sequences provided intriguing evidence for the origin of *VCY2*. The autosomal sequences contained features that disrupted the ORFs and therefore did not encode functional proteins (Fig. 4B). For example, the ATG start codon in exon 1 of *Hosa-VCY2* was replaced by ATA or ATT, while the TAA stop codon in exon 4 was substituted with AAA in both autosomal ORFs. In addition to the absence of start and stop codons, in-frame premature stop codons were also observed in the majority of the exons. The first of these was shared by both autosomal ORFs and was located in exon 1 42 nucleotides (nt) downstream of the start codon. In *VCY2*, a 4-nt insertion and a nucleotide substitution from TAA/TGA to TTA circumvented early termination. In exon 2 and exon 3 of the *Hosa-BEYLA* ORFs (a) and (b), more than six premature stop codons that were caused by nucleotide substitutions or frameshifts were detected. At least three of these were identical, for example, the downstream TAA at 133 nt and the TGA 145 nts downstream in exon 2, but none blocked the translation of *VCY2*. Four disablers (sequence differences that disrupted the ORF) were common to *Hosa-BEYLA* ORFs (a) and (b); these disablers included the absence of start and stop codons and two premature stop codons at 42 and 81 nt downstream in the ORF. The presence of these disablers suggests that the putative *Hosa-VCY2* autosomal ORFs lacked protein-coding abilities.

**Fig 5:**
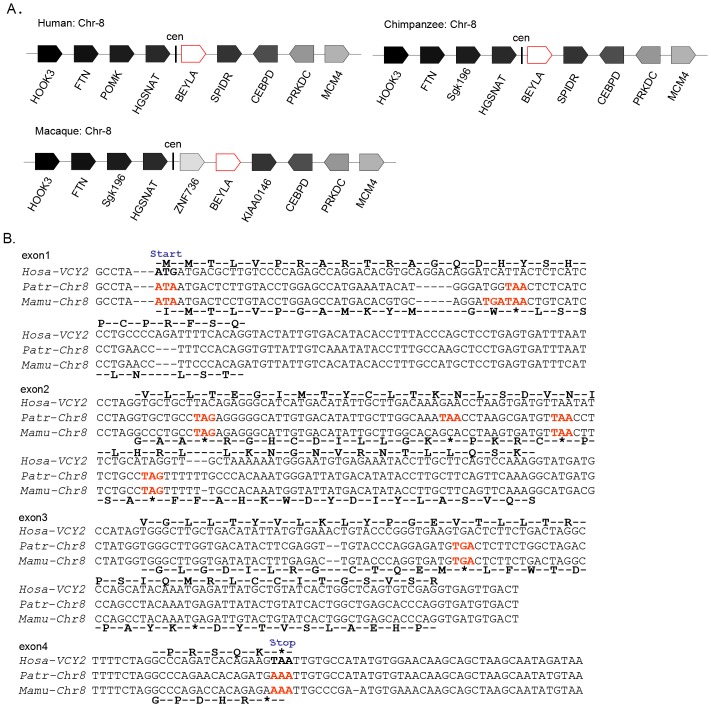
Non-protein-coding autosomal sequence homologous to *VCY2* from the autosomes of chimpanzees and rhesus macaques. (A) The synteny block comparison around human *BEYLA* and its homologous sequences from chimpanzee and rhesus macaque. The protein-coding genes were shown, the arrows indicates the orientation of each gene. (B) Sequences from chimpanzees and rhesus macaques homologous to human *VCY2* were obtained and sequences for each protein-coding exon were joined to generate a putative autosomal ORF. Artificial translations of chimpanzee *BEYLA* are shown below each row of the alignment and compared to the deduced protein sequence of *VCY2* ORF each exons above the row. Positions of start and stop codons are indicated. The asterisk (*) denotes the stop codon in the translated sequence. Disablers are indicated by red. Hosa, *Homo sapiens*; Mamu, *Macaca mulatta*; Patr, *Pan troglodytes*. Cen, centromeric end; *CEBPD*: CCAAT/enhancer binding protein (C/EBP), delta; *DHFR*: Dihydrofolate reductase; *HOOK3*: Hook microtubule-tethering protein 3; *FTNA*: farnesyltransferase, CAAX box, alpha; *HGSNAT*: Heparan-alpha-glucosaminide N-acetyltransferase; *MCM4*: Minichromosome maintenance complex component 4. *OR52E4*: Olfactory receptor family 52, subfamily E, member 4; *POMK*: protein-O-mannose kinase (synonyms of Sgk196); *PRKDC*: Protein kinase, DNA-activated, catalytic polypeptide; *SPIDR*: Scaffolding protein involved in DNA repair (*KAA0146* in rhesus macaque); *ZNF*: Zinc finger protein.

To determine whether the non-protein-coding sequences from the *Hosa-BEYLA* ORFs (a) and (b) originated from non-protein-coding primate ancestral sequences or from a pseudogene that lost its protein-coding ability, the primate autosomal *VCY2s* were examined for features suggestive of protein encoding. *Hosa-VCY2* genomic sequences were compared by BLAT against the genomic data for other primates. Fragments with similarities to *VCY2* were found in the autosomes of humans, chimpanzees and rhesus macaques; they were located near the centromeric end of Chr-8 and shared a conserved synteny block (Fig. 5A). *VCY2* autosomal sequences from four other representative primate lineages with close phylogenetic relationships with humans, including the common chimpanzee and rhesus macaque, were evaluated to clarify the evolutionary history of *VCY2*.

The autosomal sequences from Chr-8 in the chimpanzee and rhesus macaque were aligned, and the sequences with high similarities to *Hosa-VCY2* exons were used to reconstruct a putative ancestral primate autosomal ORF according to the orientation and ordering of the *Hosa-VCY2* and *BEYLA* ORFs(a) from the chimpanzee and rhesus macaque. The primate sequences were highly similar to *Hosa-BEYLA*. A comparison of the primate *BEYLA* ORF(a) and *Hosa-VCY2* ORF revealed that nearly all of the disablers in *Hosa-BEYLA* ORF(a) were retained in the primate genome, including the premature stop codons at 42 (exon 1) and 81 (exon 2) nt downstream of the start codon (Fig. 5B). Furthermore, the ATG start and TAA stop codons (exon 1 and exon 4, respectively) were also absent from the corresponding positions in all of the primate *BEYLA* sequences, which was identical to the human sequence and therefore suggested a common origin.

**Fig 6:**
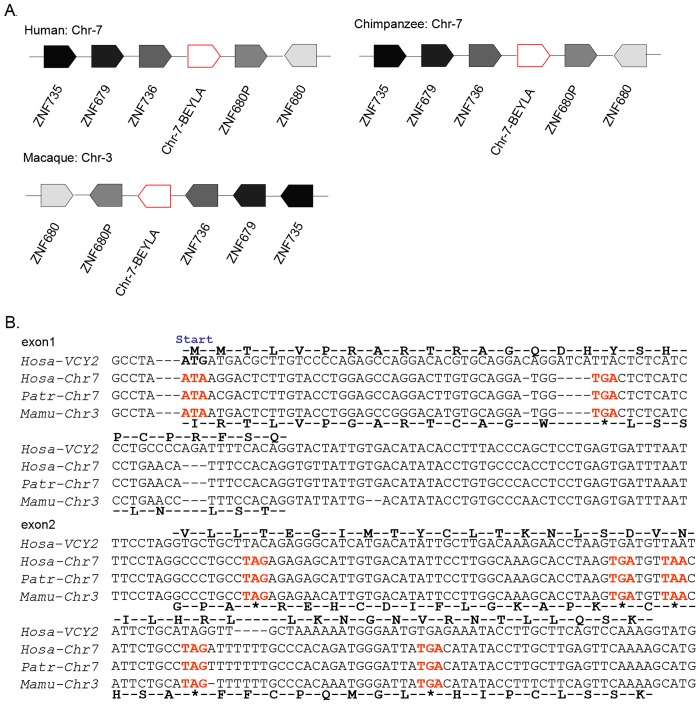
Non-protein-coding autosomal sequences homologous to *VCY2* on human Chr-7-*BEYLA* and autosomes of chimpanzees and rhesus macaques. The synteny block comparison around human *Chr-7-BEYLA* and its homologous sequences from chimpanzee and rhesus macaque. The protein-coding genes were shown, the arrows indicates the orientation of each gene. Fragments homologous to *VCY2* were extracted from human, chimpanzee Chr-7 and rhesus macaque Chr-3. Protein-coding exons were joined to obtain the putative autosomal *VCY2* ORF. (B) Artificial translations of Chr-7-*BEYLA* ORF(a) exon 1 and exon 2 are shown below each row of the alignment and compared to the deduced protein sequence for *VCY2* ORF above the row. Positions of start and stop codons are indicated. Disablers are indicated by red. Hosa, *Homo sapiens*; Mamu, *macaca mulatta*; Patr, *Pan troglodytes*; *ZNF*: Zinc finger protein.

In addition to Chr-8, we also found genomic fragments that exhibited high similarities with *VCY2* in the chimpanzee (Chr-7) and rhesus macaque (Chr-3). This region spanned 5 kb and was located in a zinc finger (*ZNF*) gene cluster. The neighboring genes included *ZNF680*, *ZNF736*, *ZNF735* and *ZNF679*. Gene synteny analyses centered on Chr-7-*BEYLA* and Chr-3-*BEYLA* from the human, chimpanzee and rhesus macaque revealed a conserved synteny block. The orders and orientations of the *ZNF* clusters were the same in these three species, which supports the homologous relationship of this region (Fig. 6A). Only two potential protein-coding exons were identified, and these were confirmed as being non-protein-coding in the alignment due to the presence of identical disablers that included the lack of a start codon and the presence of a premature stop codon (Fig. 6B).

A similar observation was made for the other primate *BEYLA* ORF, in which the disablers (i.e., no start or stop codons) were primarily in autosomal sequences. We showed the autosomal sequences from great apes like gorillas (S2 Fig.) and bonobos (S3 Fig.), gibbons (S4 Fig.), Old World monkeys such as baboons (S5 Fig.) and the Western African sabaeus monkeys (S6 Fig.). These results indicate that the primate sequences of the autosomal *VCY2* were also non-protein-coding; the sequence conservation, including the presence of the disablers, suggests a common non-protein-coding ancestral gene that predates the autosome-to-Y transposition.

### A non-protein-coding autosomal *VCY2* homolog in the marmoset

**Fig 7:**
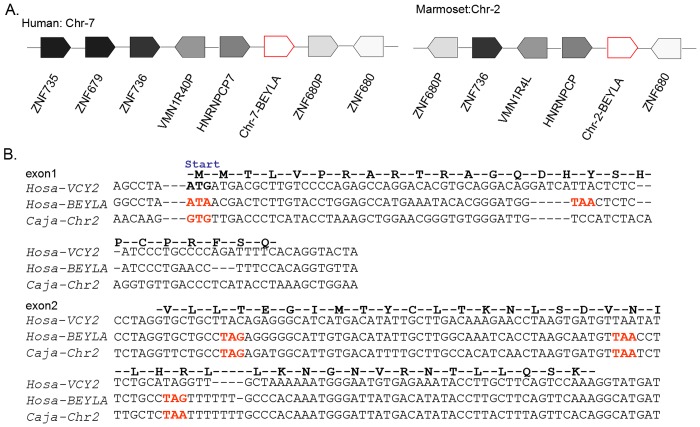
Non-protein-coding autosomal sequences homologous to *VCY2* on marmoset Chr-2. (A) The synteny block comparison around human *Chr-7-BEYLA* and its homologous sequences from marmoset Chr-2. The arrows indicates the orientation of each genes. Fragments homologous to *VCY2* extracted from human were used to compare with the fragments from marmoset. Protein-coding exons were joined to obtain the putative autosomal *VCY2* ORF. (B) The deduced protein sequence for *VCY2* ORF was shown above the row. Positions of start codons are indicated. Disablers are indicated by red. Caja, *Callithrix jacchus*; Hosa, *Homo sapiens*.

To exclude the possibility that the autosomal locus *BEYLA* had deteriorated from a protein-coding into a non-protein-coding gene, the status of *BEYLA* in the out-group species was examined. The green amplicon was duplicated and transposed into Chr-Y in the common ancestor of Old World monkeys and apes after the separation of the New World monkeys. We used the genome of the common marmoset (*Callithrix jacchus*), which is a New World monkey that has been fully sequenced and annotated, to clarify the possible ancestral state of *VCY2* before transposition. First, we confirmed that *VCY2* was absent from the marmoset Chr-Y. According to the BLAT results, the marmoset Chr-Y genomic sequences also did not contain any regions that exhibited significant sequence similarities with the human *VCY2*. Neither the sequence from the marmoset testis EST clone library nor the testis transcriptome data exhibited significant sequence similarities with the *VCY2* mRNA or genomic sequences (Table 3). Subsequently, in the autosomal genomic sequences, we found that a genomic region in Chr-2 of approximately 30 kb exhibited sequence similarities with both *VCY2* and *BEYLA*. Only two potential protein-coding exons were identified, and these were confirmed to be being non-protein-coding in the alignment (Fig. 7B). These results confirm that the marmoset autosomal *VCY2* in Chr-2 might not be protein-coding because the ‘ATG’ start codon was replaced by ‘GTG’. The results of a synteny analysis of the region centered on *VCY2* and the human Chr-7 *BEYLA* are shown in Fig. 7A. The *ZNF736*, *ZNF680*, heterogeneous nuclear ribonucleoprotein C pseudogene (*HNRNPCP*) and vomeronasal receptor pseudogene (*VN1RP*) were located in both human Chr-7 and marmoset Chr-2. The synteny analysis convinced us that this region is the homologous sequences of the human *VCY2* autosomal genomic sequences. These data support the notion that *VCY2* might have been derived from a non-protein-coding region.

### Non-protein-coding *VCY2* from rhesus macaque Chr-Y

**Fig 8:**
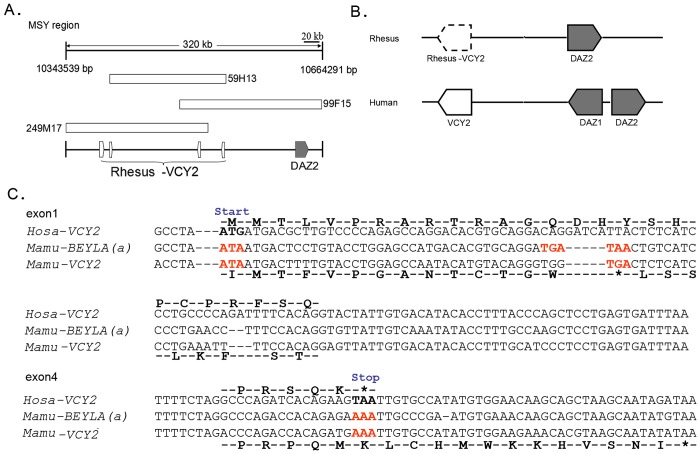
Non-protein-coding *VCY2* on the rhesus macaque Chr-Y male-specific region. (A) Homologous fragments of *VCY2* from three overlapping BAC clones (CH250–99F15, CH250–59H13, and CH250–249M17) were extracted from the rhesus macaque male-specific region of Chr-Y (MSY). The position of each BAC clone in the whole MSY region is marked. (B) A putative synteny block around the *VCY2* from rhesus macaque was reconstructed and compared with the synteny block of human *VCY2*. (C) Fragments homologous to *VCY2* were extracted from rhesus macaque Chr-8 and Chr-Y. Artificial translations of rhesus macaque *VCY2* exon 1 and exon 4 are shown below each row of the alignment and compared to the deduced protein sequence of the *VCY2* ORF above the row. Positions of start and stop codons are indicated. Disablers are indicated by red. *DAZ*, deleted in the azoospermia protein; Hosa, *Homo sapiens*; Mamu, *Macaca mulatta*.

A previous study showed the *VCY2* gene located in the green amplicon was generated by duplication and transposition of autosomal segments before the ape-monkey split [38] and that no homolog is present in the Old World monkey Chr-Ys such as that of the rhesus macaque [42]. The absence of a *VCY2* protein-coding translation in the rhesus macaque was confirmed using the *Hosa-VCY2* cDNA sequence in a BLAST search against the rhesus macaque testis cDNA (SRA039857). We also searched the database using the genomic sequences of *VCY2* and found a rhesus testis cDNA that exhibited sequence similarities with human *VCY2* (identities over 75% and 460 bp lengths). To investigate the events that occurred after the autosome-to-Y transposition, rhesus *MSY* BAC clones were searched for regions with high sequence similarity to *VCY2*. Three overlapping BAC clones (CH250–99F15, CH250–59H13, and CH250–249M17) close to the centromere were identified and are referred to as a *VCY2* homolog (Fig. 8A).

Rhesus macaque *VCY2* was located in the DAZ cluster in the rhesus macaque, and a DAZ homolog was found in the 5' neighboring region (Fig. 8B). According to the evolutionary history of the Chr-Y AZFc region, the green amplicon (presumably containing *VCY2*) likely emerged in Chr-Y via an ancestral autosomal transposition that occurred prior to the ape-monkey-split and also included the neighboring red amplicon with the autosomal transposed ancestral *DAZ* gene from Chr-3. These findings are consistent with the observations from the Chr-Y of the rhesus macaque. In humans, *VCY2* is also located within the *DAZ1*/*DAZ2* gene cluster, which reflects their orthologous relationship in evolutionary history. The *DAZ* gene duplication is also consistent with this evolutionary history. The alignments of the *Hosa-VCY2* ORF and the sequences with similarities in the rhesus macaque revealed divergent sequences, and the majority of the disablers that were observed in the primate autosome appeared in the rhesus macaque Chr-Y *VCY2*. The artificial translation of rhesus macaque *VCY2* revealed disrupted ORFs, and the same disablers that were present in the primate autosomal sequences, including the lack of start and stop codons (ATA and AAA, respectively) and several premature stop codons (Fig. 8C), were also observed, which reflects the status of the gene shortly after non-protein-coding autosomal segment transposition. The autosomal homologous sequence was maintained as a non-protein-coding region in all primate lineages, which supports the hypothesis that *de novo* protein-coding genes can originate from transposed, non-protein-coding autosomal segments on Chr-Y.

### Non-protein-coding pseudogene *BPY2DP*

Pseudogenes are genomic DNA sequences that are similar to normal genes but are non-functional; they are regarded as defunct relatives of functional genes. The majority of pseudogenes are dysfunctional relatives of genes that have lost their protein-coding ability. However, regarding possible *de novo-*originated protein-coding genes, such pseudogenes might be dysfunctional protein-coding genes but might also be ancestral copies prior to obtaining protein-coding abilities. Human *BPY2DP* is a pseudogene of *VCY2* that is located in the Yp11.2 region, while all other protein-coding *VCY2* paralogs are located in Yq11 (Table 1). The synteny block around the *BPY2DP* exhibited a situation very similar to that of the green amplicon. Green-ampliconic genes, such as TTTY17, *TRIM60P*, and *ZNF736P*, are located on both side of the synteny block (S7A Fig.). These findings might imply that *BPY2DP* was created through an amplicon duplication. The dot-plot matrix revealed the sequences similarities between the genomic sequences of human *BPY2DP* and *VCY2* (S7B Fig.). Subsequently, we examined the sequence differences between *BPY2DP* and *VCY2* to clarify why *BPY2DP* did not have a protein-coding ability. According to the sequence alignment, although *BPY2DP* had obtained the start and stop codons, it also had some conserved disablers, such as the premature stop-codon in exon 1 (S7C Fig.). We supposed that *BPY2DP* might be a non-protein-coding ancestral copy that existed prior to the *VCY2* obtaining protein-coding ability, but we needed more information from other primate Chr-Y to support this hypothesis.

### Duplicated segments in the 5’-untranslated region

The dot-plot matrix of the *VCY2* genomic sequence revealed an 8.5-kb segment in the *VCY2* 5’-untranslated region (5’-UTR) region that exhibited significant sequence similarities with the protein-coding region of *VCY2* (S8A Fig.). This region was located approximately 2 kb upstream of the *VCY2* translation start. We also found a similar situation in all of the 5’-UTR regions of the human *VCY2* paralogs, including the pseudogene *BPY2DP*. Similarly, a duplicated segment in the 5’-UTR of the chimpanzee *VCY2* homolog was also identified. Subsequently, we aligned both the human and chimpanzee *VCY2*–5’-UTR and *VCY2*-ORF genomic region sequences and compared the sequence differences. Surprisingly, the sequence disablers that we found in the human and chimpanzee *VCY2*–5’-UTR regions were nearly identical to those in the autosomal *BEYLA* (S8B Fig.). For example, both the start and stop codons were absent, and pre-mature stop codons prevented further translation. These sequence disablers obstructed the ORF. None of the exons from the human *VCY2* coding region matched the 5’-UTR exons; thus, if these two segments were generated through a duplication of one identical copy, we suppose that the exon and intron structures of *VCY2* might have been rearranged and changed after that event. The *5’-*UTR contained key elements for the regulation of gene translation, and it is possible that this 5’-UTR duplicated segment might also have a role in the regulation of the expression of *VCY2*.

### Reconstructing the evolutionary history of *VCY2* in primates

**Fig 9:**
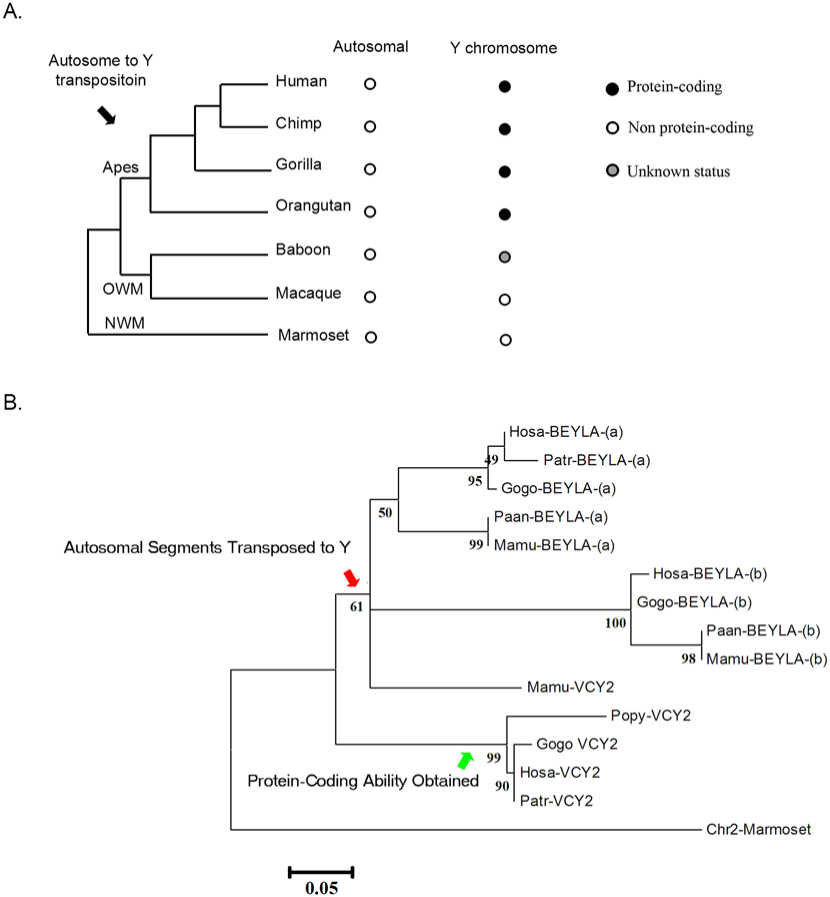
Reconstruction of the evolutionary history of *VCY2*. (A) The protein-coding ability of *VCY2* and its homologs in seven representative primate autosomes or Chr-Y in this study. The lineage relationship among these species was referred to the Ensembl database (www.Ensembl.org/). (B) Phylogenetic analysis of the *VCY2* gene and its homologs in primates. The phylogenetic tree was constructed based on the 84 nt around *VCY2* protein-coding exon 1 and from autosomal *BEYLA* using the Maxium likelihood method. Bootstrap values for 500 replicates are shown next to branches; branch lengths are indicated in the same units as the evolutionary distances used to construct the phylogenetic tree. Critical evolutionary events for *VCY2* (red and green arrows) and the origin of different clades (square brackets) are indicated. Caja, *Callithrix jacchus*; Gogo, *Gorilla gorilla*; Hosa, *Homo sapiens*; Mamu, *Macaca mulatta*; NWM, New World monkeys; OWM, Old World monkeys; Paan, *Papio anubis*; Patr, *Pan troglodytes*; *Popy*, *Pongo pygmaeus*.

The relationships between *VCY2* and its protein-coding and non-protein-coding homologs from the Chr-Y or autosomes of human, apes, Old World monkeys and New World monkeys were established based on the evolutionary history of primates (Fig. 9A). The disablers are common to the human *BEYLA*, primate autosomal *BEYLA*, rhesus macaque *VCY2* and marmoset Chr-2. These non-protein-coding sequences with common disablers were indicative of an autosomal non-protein-coding ancestral sequence. The *VCY2* homolog protein-coding gene was absent in the marmoset Chr-Y, and the autosomal homolog was non-protein-coding. *VCY2* was found in the Chr-Y of the rhesus macaque, while *VCY2* mRNA expression was confirmed in the orangutan, gorilla, chimpanzee and human, which implies that the protein-coding ability was acquired after the ape-monkey split. To illustrate the evolutionary history, a phylogenetic tree for *VCY2* (exon 1) and its homologs was constructed using the maximum likelihood (ML) method. The *VCY2* homologous sequences from marmoset Chr-2 formed the out-group of the tree. The topological structure of the in-group exhibited two major clades; i.e., *VCY2* and *BEYLA* (Fig. 9B). Interestingly, the rhesus macaque *VCY2* was closely related to the autosomal *BEYLA*, which might suggest a recent transposition event. The human, chimpanzee, gorilla and orangutan *VCY2s* formed a unique clade, and their evolutionary relationship implies that the protein-coding ability might have been obtained after the divergence of Old World monkeys and apes.

Considering the above findings, these data suggest a model for the origin of *VCY2* in which the autosomal, non-protein-coding homolog was duplicated and transposed to the Chr-Y before the ape-monkey split at least 35 mya and was retained in the rhesus macaque as a non-protein-coding gene. *VCY2* acquired its protein-coding ability before the monkey-ape split.

## Discussion

Mammalian Chr-Y genes play vital roles in sex determination, spermatogenesis, and other male-specific processes [65,66]. The evolutionary histories and origins of the majority of Chr-Y genes have been extensively studied, but *de novo* Chr-Y protein-coding genes have never been reported. Chr-Y evolved from an ancestral autosome, and it has a unusual evolutionary history [67–69]. Chr-Y translocation and segmental duplication have been common in both humans and fruit flies, and the accumulation of transposable elements on the Chr-Y during its evolution has been observed on both the *Drosophila* and human Chr-Y [70,71]. Autosome-to-Y transposition of male-benefit genes is also common and conserved in mice [72], carnivores [73] and bovines [74]. Male-benefit genes such as *DAZ* have been accumulated on the Chr-Y through transposition and retroposition from autosomes and subsequent amplification [38, 39]. However, to our knowledge, this is the first report of a possible *de novo* autosome-to-Y transposed and amplified protein-coding gene. *VCY2* is frequently deleted in patients with idiopathic infertility; thus, *VCY2* is likely to function in male germ cell development and to be involved in the pathogenesis of male infertility [62]. The evidence we presented here regarding the origin of *VCY2* suggests that *de novo* protein-coding genes can be generated through the transposition of ancestral autosomal non-protein-coding segments. This process might explain the birth of *VCY2* as a gene since it might help to improve the male fertility.

Recent studies have revealed that the life cycle of a *de novo* gene is a multi-step process [8,11]. In yeast models, functional genes evolve *de novo* through putative transitory proto-genes. Our results also provided some clues about these processes. Our evidence also suggests that *VCY2* was not transformed into a protein-coding gene immediately after transposition because the *VCY2* protein-coding homolog is absent in the rhesus macaque, but the non-protein-coding genomic sequence is present. These findings might imply that the *VCY2* stayed as a non-protein-coding gene shortly after autosome-to-Y transposition. The protein-coding ability of *VCY2* might have been gained after the ape-monkey split because mRNA evidence was found in several species of apes. The non-protein-coding segments in the 5’-UTR are indicative of a duplication event inside the ancestral *VCY2* genomic region before the protein-coding ability was obtained. The presence of a pseudogene (*BPY2DP*) might suggest that an amplicon duplication event occurred before *VCY2* obtained its protein-coding ability. This evidence suggests that the *de novo* origination of *VCY2* might be much more complicated than we expected.

Previous study revealed that long non-coding RNAs, especially those with active and regulated transcription, may serve as a birth pool for protein-coding genes and 24 hominoid-specific novel protein-coding genes originating from long non-protein-coding RNAs [22] have also been identified. Our results provide an other unique example for this model. The autosomal loci (*BEYLA*) is a long intergenic non-protein coding RNA in human and primate genomes. The homologous sequences from all of the other primates, including marmosets, consist of non-protein-coding genes, which indicates that the ancestral autosomal loci was non-protein-coding. Interestingly, EST clones from the testis tissues matched this gene, and the expression profile from the NONCODE database also exhibited a strong preference in testis tissue. These clues imply a possible role of *BEYLA* in male-specific functions.

*De novo* protein-coding genes might be generated from non-protein-coding genomic sequences, and such genetic material might be derived in different manners. The present study also provides the first example of a possible *de novo* protein-coding gene that was generated from duplicated and transposed segments. Segmental duplication has frequently occurred during primate evolution, and it is estimated that approximately 5%–10% of human genomic sequences were generated through this process [75]. Whether these duplicated regions contain any *de novo* protein-coding genes is a question that warrants further investigation.

The emergence of novel genes is a driving force for evolutionary innovation in all organisms, and the acquisition of novel gene functions is important for species-specific adaptations. *VCY2* is a testis-specific gene in the *AZFc* region that is frequently deleted in infertile patients with Yq microdeletions [76], which leads to defective spermatogenesis and an increased risk of infertility but not essential [77–79]. VCY2 binding partners include the ubiquitin-protein ligase E3A and the microtubule-associated protein-like protein MAPS1, which suggest that *VCY2* interacts with evolutionarily ancient genes. These lines of evidence suggest that this novel gene has found binding partners and has new functions.

## Conclusions

In conclusion, the evidence presented here supports the emergence of a protein-coding function in a Chr-Y gene that originated from a transposed autosomal segment. These findings provide novel insights into the mechanism of gene creation and the evolutionary history of the Chr-Y.

## Supporting Information

S1 Fig The alignments of the flanking sequences of human *VCY2* with the autosomal sequences from Chr-8 (*BELYA*) and Chr-7. The 200 bp flanking sequences from upstream (A) or downstream (B) of *VCY2* gene are aligned with the autosomal sequences from *BEYLA* (Chr-8) and Chr-7. The regions for the coding sequence (CDS) of *VCY2* are shown as gray. Positions of start and stop codons are indicated. Disablers are indicated by red. The translations of human *VCY2* coding sequences are shown above each row of the alignment.
(TIF)

S2 Fig Non-protein-coding autosomal sequences homologous to *VCY2* from gorilla autosomes. The synteny block comparison around human Chr-8 *BEYLA* (A) and Chr-7 (B) and its homologous sequences from gorilla Chr-8 and Chr-7. (C) Gorilla autosomal sequences were extracted and aligned with human *VCY2* and *BEYLA*. The deduced protein sequence of *VCY2* exon 1 and exon 4 were shown above the row. Positions of start and stop codons are indicated. The asterisk (*) denotes the stop codon in the translated sequence. Disablers are indicated by red. Gogo, *Gorilla gorilla*; Hosa, *Homo sapiens*.
(TIF)

S3 Fig Non-protein-coding autosomal sequences homologous to *VCY2* from bonobo autosomes. The synteny block comparison around human Chr-8 *BEYLA* (A) and Chr-7 (B) and its homologous sequences from bonobo genomes (unplaced scaffolds: NW_003870542.1 and NW_003870421.1). (C) Bonobo sequences were extracted and aligned with human *VCY2* and BEYLA. The deduced protein sequence of *VCY2* exon 1 and exon 4 were shown above the row. Positions of start and stop codons are indicated. The asterisk (*) denotes the stop codon in the translated sequence. Disablers are indicated by red. Hosa, *Homo sapiens*; Papa, *Pan paniscus*.
(TIF)

S4 Fig Non-protein-coding autosomal sequences homologous to *VCY2* from gibbon autosomes. The synteny block comparison around human Chr-7 (B) and its homologous sequences from gibbon Chr-17. (C) Gibbon autosomal sequences were extracted and aligned with human *VCY2* and *BEYLA*. The deduced protein sequence of *VCY2* exon 1 was shown above the row. Positions of start and stop codons are indicated. The asterisk (*) denotes the stop codon in the translated sequence. Disablers are indicated by red. Hosa, *Homo sapiens*; Nole, *Nomascus leucogenys*.
(TIF)

S5 Fig Non-protein-coding autosomal sequences homologous to *VCY2* from baboon autosomes. The synteny block comparison around human Chr-8 *BEYLA* (A) and Chr-7 (B) and its homologous sequences from baboon Chr-8 and Chr-3. (C) Baboon autosomal sequences were extracted and aligned with human *VCY2* protein-coding exons. The deduced protein sequence of *VCY2* exon 1 and exon 4 were shown above the row. Positions of start and stop codons are indicated. The asterisk (*) denotes the stop codon in the translated sequence. Disablers are indicated by red. Hosa, *Homo sapiens*; Paan, *Papio anubis*.
(TIF)

S6 Fig Non-protein-coding autosomal sequences homologous to *VCY2* from the West African sabaeus monkey autosomes. The synteny block comparison around human Chr-8 *BEYLA* (A) and Chr-7 (B) and its homologous sequences from the West African sabaeus monkey Chr-8 and Chr-3. (C) West African sabaeus monkey autosomal sequences were extracted and aligned with human *VCY2* and *BEYLA*. The deduced protein sequence of *VCY2* exon 1 and exon 4 were shown above the row. Positions of start and stop codons are indicated. The asterisk (*) denotes the stop codon in the translated sequence. Disablers are indicated by red. Chsa, *Chlorocebus sabaeus*; Hosa, *Homo sapiens*.
(TIF)

S7 Fig The protein-coding ability of pseudogene *BPY2DP*. The synteny block comparison around human pseudogene gene *BPY2DP* and *VCY2* (*BPY2*). The order and orientation for each gene in the green-amplicon was shown. (B) The dot-plot analysis for *BPY2DP* and *VCY2* genomic sequences. Each dot in the map represent a fragment of 14 bp length (mismatch is 0) in the alignment. (C). The alignment of the *BPY2DP* and partial protein-coding sequences of *VCY2*, the deduced protein sequence for *VCY2* exon 1 and exon 4 above the row. Positions of start codons are indicated. Disablers are indicated by red. Red and green arrow indicate the enabler and disabler for *BPY2DP*. Hosa, *Homo sapiens*
(TIF)

S8 Fig Non-protein-coding duplicated segments in human and chimpanzee *VCY2* 5’-untranslated region. The dot-plot analysis of the human *VCY2* genomic sequence with itself. Each dot in the map represent a fragment of 11 bp length (mismatch is 0) in the alignment. The exon-intron structure of human *VCY2* was shown. The position of protein-coding exons of *VCY2* were indicated as 1–4. The protein-coding exons (black bars) and non-protein-coding exons in 5’-UTR and 3’UTR (white bars); 1 cm in the diagram = 2 kb in the genomic sequence. The arrow and the red dash lines indicated the positions of the duplicated segments (B) Artificial translations of-*VCY2* 5’UTR ORF are shown below each row of the alignment and compared to the deduced protein sequence for *VCY2* ORF above the row. Positions of start and stop codons are indicated. Disablers are indicated by red. Hosa, *Homo sapiens*; Patr, *Pan troglodytes*; UTR, untranslated region.
(TIF)

S1 Table The versions of genomic assemblies referred to and the sequence information used in this article. (XLS)

S2 Table The bacterial artificial chromosome (BAC) clones of rhesus macaque Y chromosome male-specific region referred to in this article and the sequence information. (XLS)

S3 Table The human autosomal fragments longer than 4 kilobase pairs that exhibits high sequence similarities with human *VCY2*. (XLS)

S4 Table The expression profiles of human *BEYLA* from non-coding RNA (NONCODE) database derived from RNA-seq expression analysis. (XLS)
