# Supplementary figures and images for: De Novo Origin of VCY2 from Autosome to Y-Transposed Amplicon
Source: PLoS One. 2015 Mar 23;10(3):e0119651. doi: 10.1371/journal.pone.0119651 (PMC4370482; doi:10.1371/journal.pone.0119651)

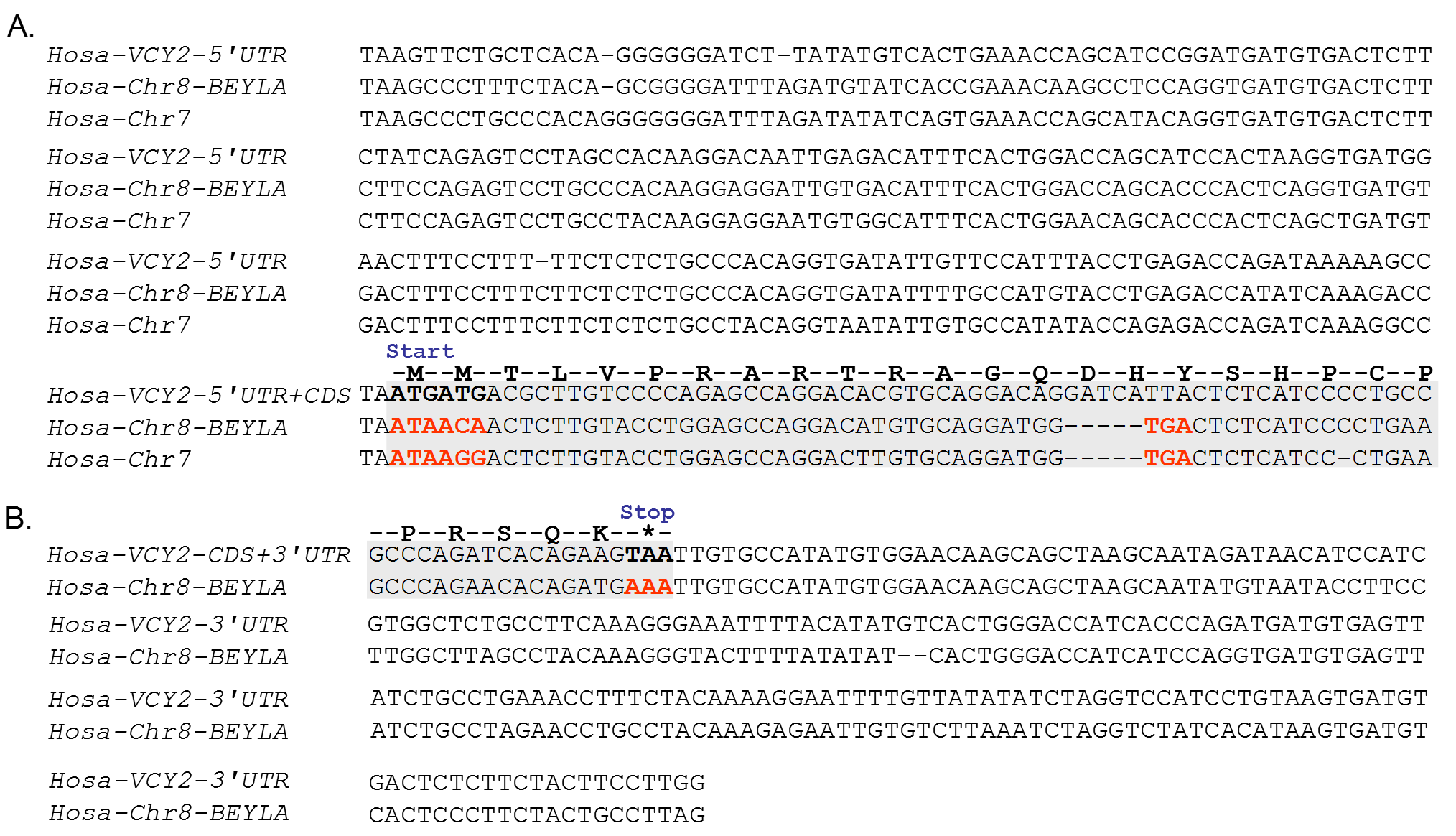

Supplement: S1 Fig — The 200 bp flanking sequences from upstream (A) or downstream (B) of VCY2 gene are aligned with the autosomal sequences from BEYLA (Chr-8) and Chr-7. The regions for the coding sequence (CDS) of VCY2 are shown as gray. Positions of start and stop codons are indicated. Disablers are indicated by red. The translations of human VCY2 coding sequences are shown above each row of the alignment. (TIF) [file pone.0119651.s001.tif]

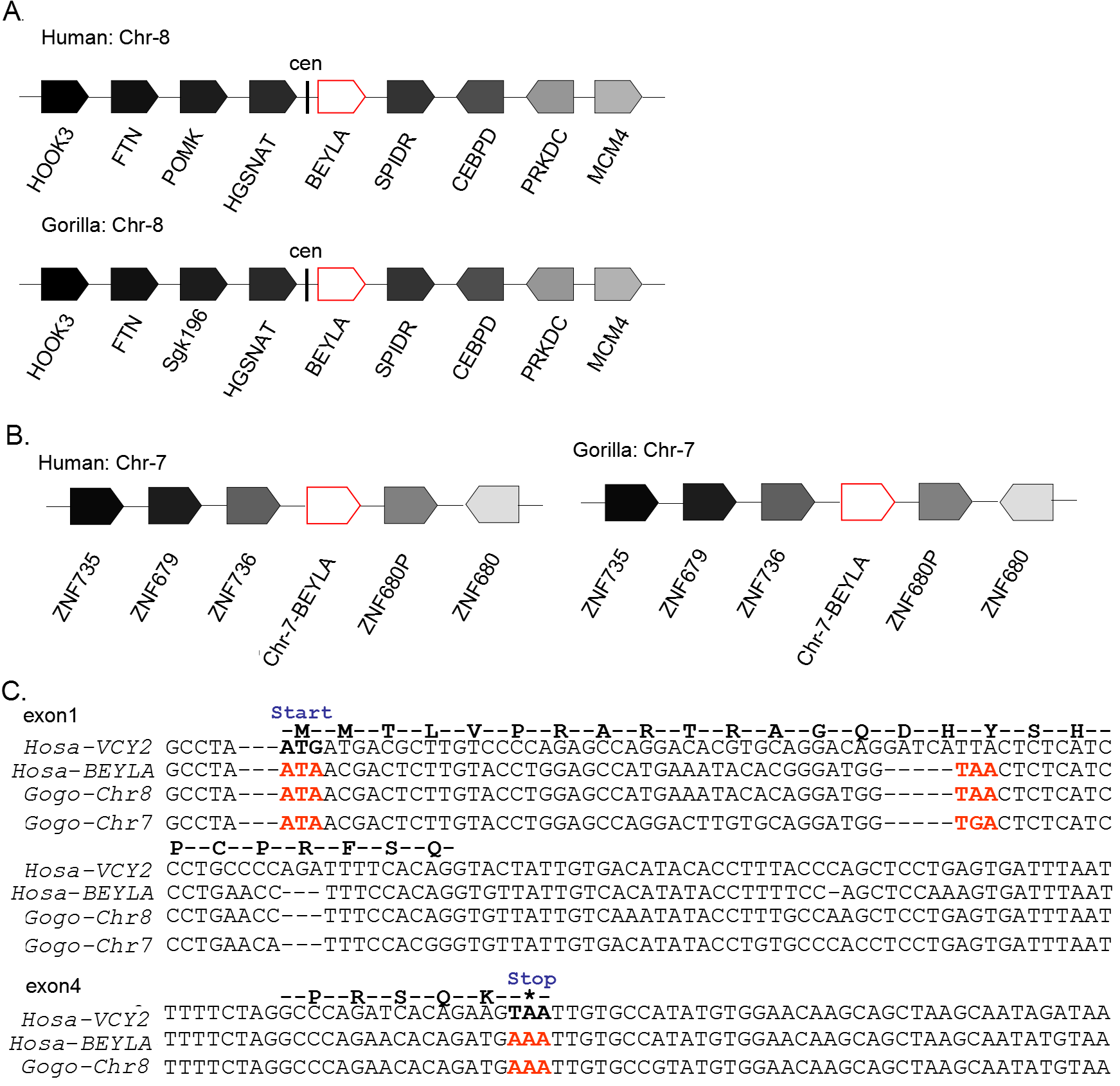

Supplement: S2 Fig — The synteny block comparison around human Chr-8 BEYLA (A) and Chr-7 (B) and its homologous sequences from gorilla Chr-8 and Chr-7. (C) Gorilla autosomal sequences were extracted and aligned with human VCY2 and BEYLA. The deduced protein sequence of VCY2 exon 1 and exon 4 were shown above the row. Positions of start and stop codons are indicated. The asterisk (*) denotes the stop codon in the translated sequence. Disablers are indicated by red. Gogo, Gorilla gorilla; Hosa, Homo sapiens. (TIF) [file pone.0119651.s002.tif]

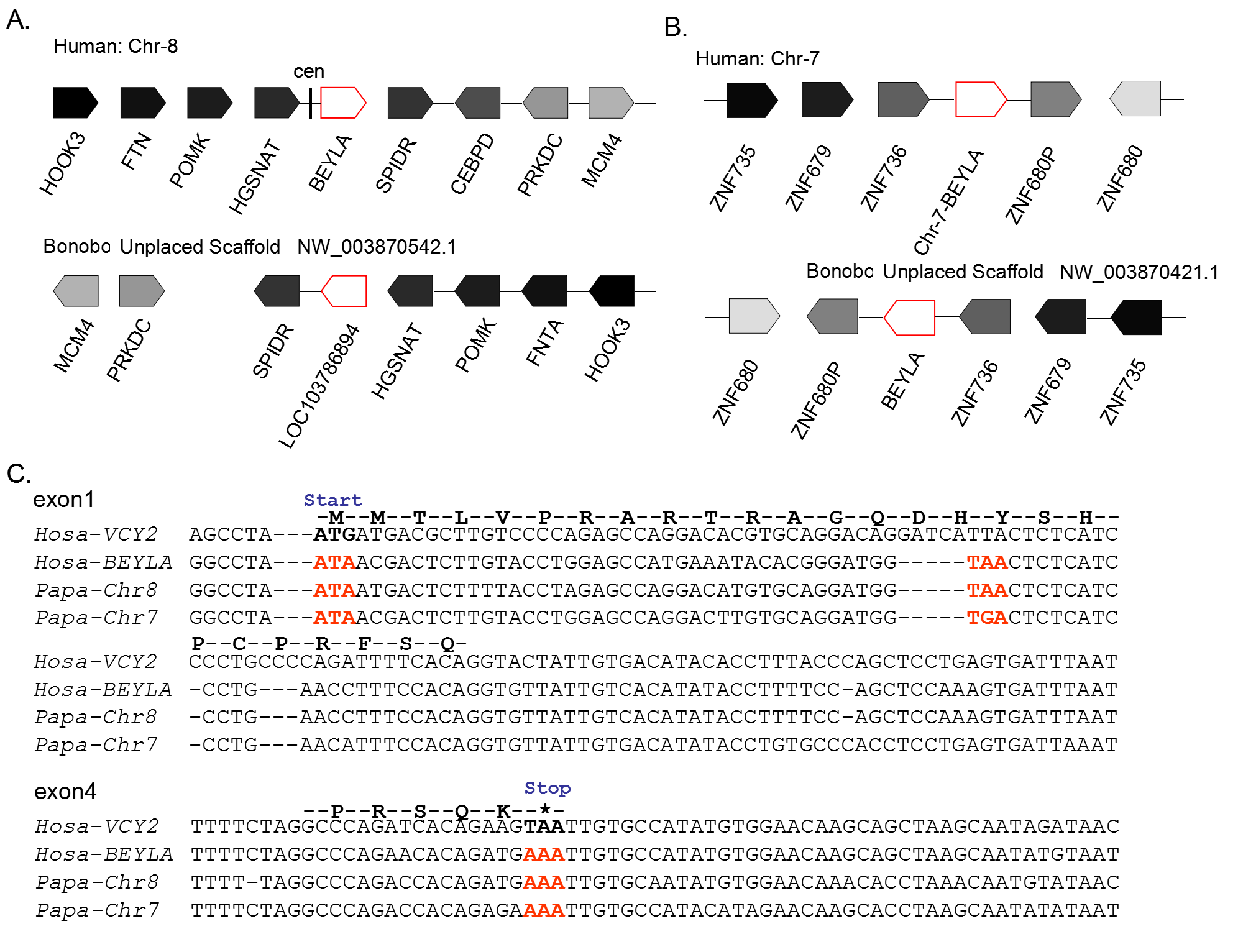

Supplement: S3 Fig — The synteny block comparison around human Chr-8 BEYLA (A) and Chr-7 (B) and its homologous sequences from bonobo genomes (unplaced scaffolds: NW_003870542.1 and NW_003870421.1). (C) Bonobo sequences were extracted and aligned with human VCY2 and BEYLA. The deduced protein sequence of VCY2 exon 1 and exon 4 were shown above the row. Positions of start and stop codons are indicated. The asterisk (*) denotes the stop codon in the translated sequence. Disablers are indicated by red. Hosa, Homo sapiens; Papa, Pan paniscus. (TIF) [file pone.0119651.s003.tif]

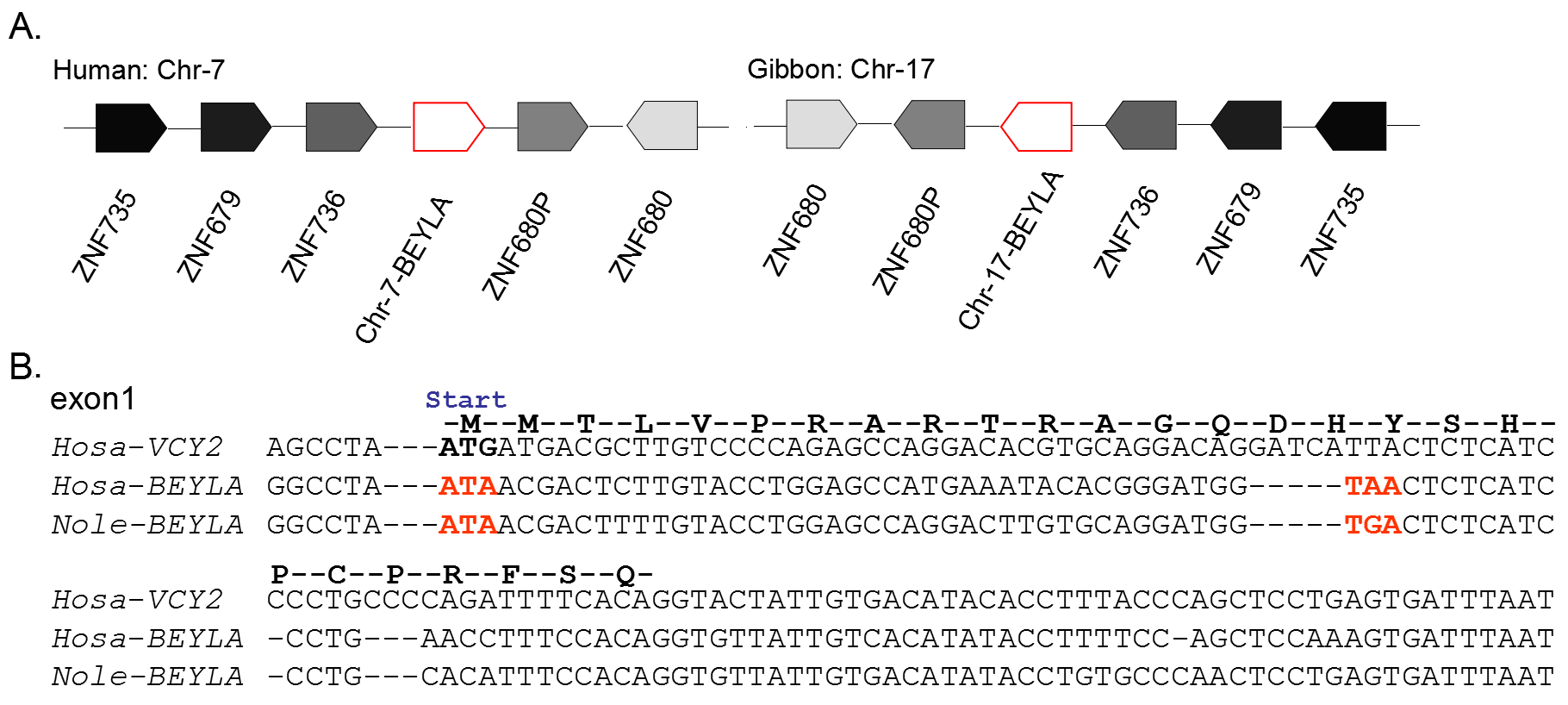

Supplement: S4 Fig — The synteny block comparison around human Chr-7 (B) and its homologous sequences from gibbon Chr-17. (C) Gibbon autosomal sequences were extracted and aligned with human VCY2 and BEYLA. The deduced protein sequence of VCY2 exon 1 was shown above the row. Positions of start and stop codons are indicated. The asterisk (*) denotes the stop codon in the translated sequence. Disablers are indicated by red. Hosa, Homo sapiens; Nole, Nomascus leucogenys. (TIF) [file pone.0119651.s004.tif]

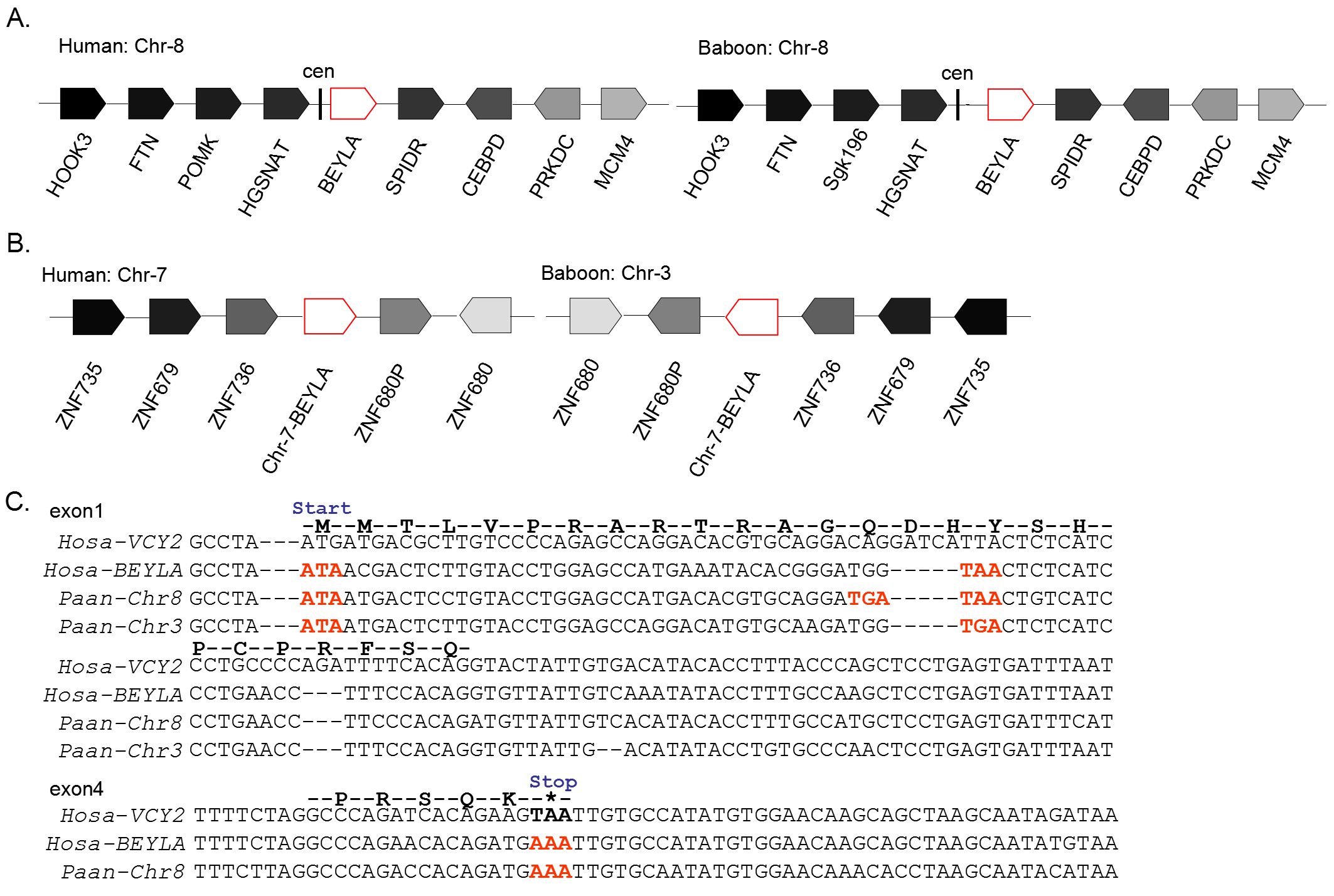

Supplement: S5 Fig — The synteny block comparison around human Chr-8 BEYLA (A) and Chr-7 (B) and its homologous sequences from baboon Chr-8 and Chr-3. (C) Baboon autosomal sequences were extracted and aligned with human VCY2 protein-coding exons. The deduced protein sequence of VCY2 exon 1 and exon 4 were shown above the row. Positions of start and stop codons are indicated. The asterisk (*) denotes the stop codon in the translated sequence. Disablers are indicated by red. Hosa, Homo sapiens; Paan, Papio anubis. (TIF) [file pone.0119651.s005.tif]

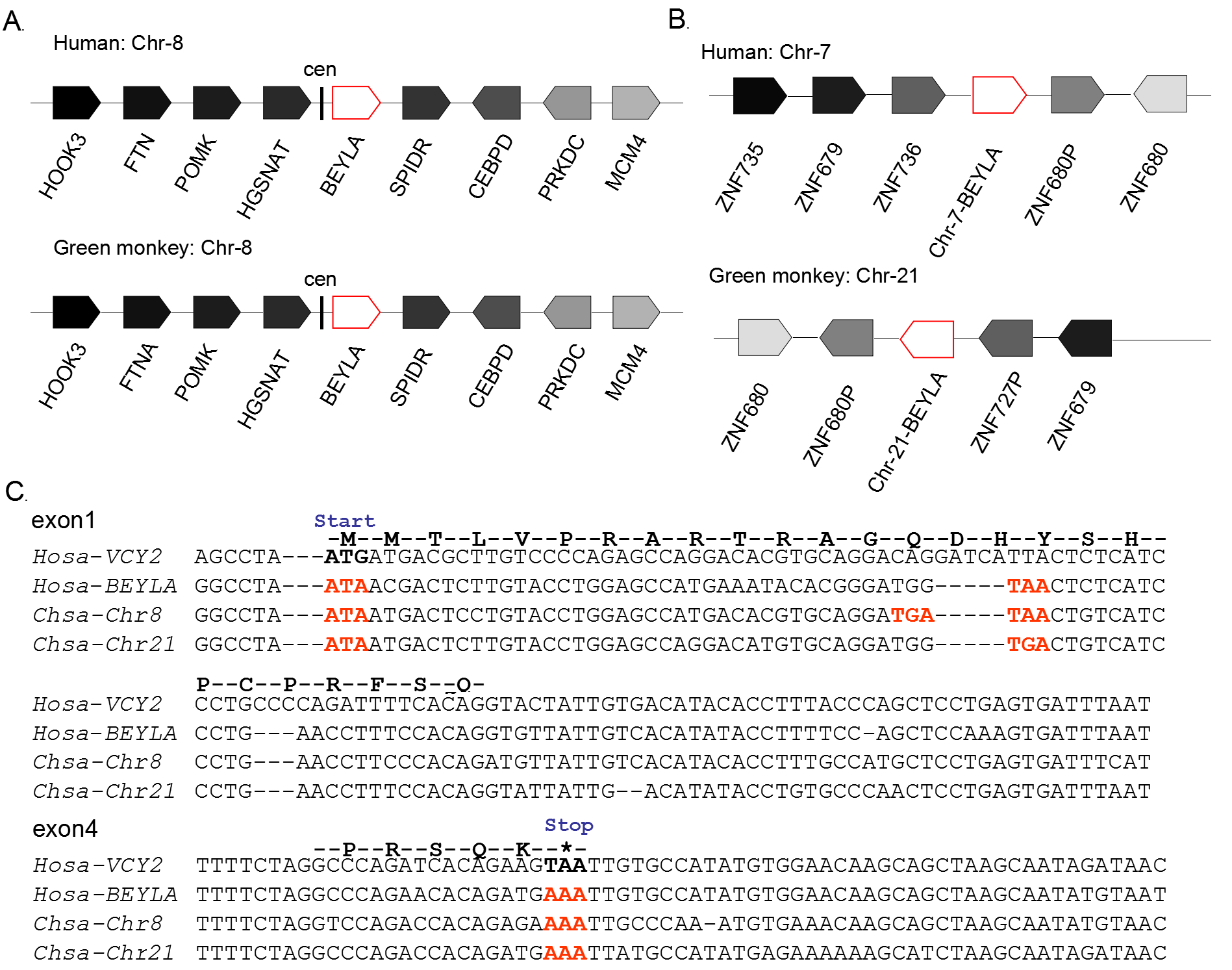

Supplement: S6 Fig — The synteny block comparison around human Chr-8 BEYLA (A) and Chr-7 (B) and its homologous sequences from the West African sabaeus monkey Chr-8 and Chr-3. (C) West African sabaeus monkey autosomal sequences were extracted and aligned with human VCY2 and BEYLA. The deduced protein sequence of VCY2 exon 1 and exon 4 were shown above the row. Positions of start and stop codons are indicated. The asterisk (*) denotes the stop codon in the translated sequence. Disablers are indicated by red. Chsa, Chlorocebus sabaeus; Hosa, Homo sapiens. (TIF) [file pone.0119651.s006.tif]

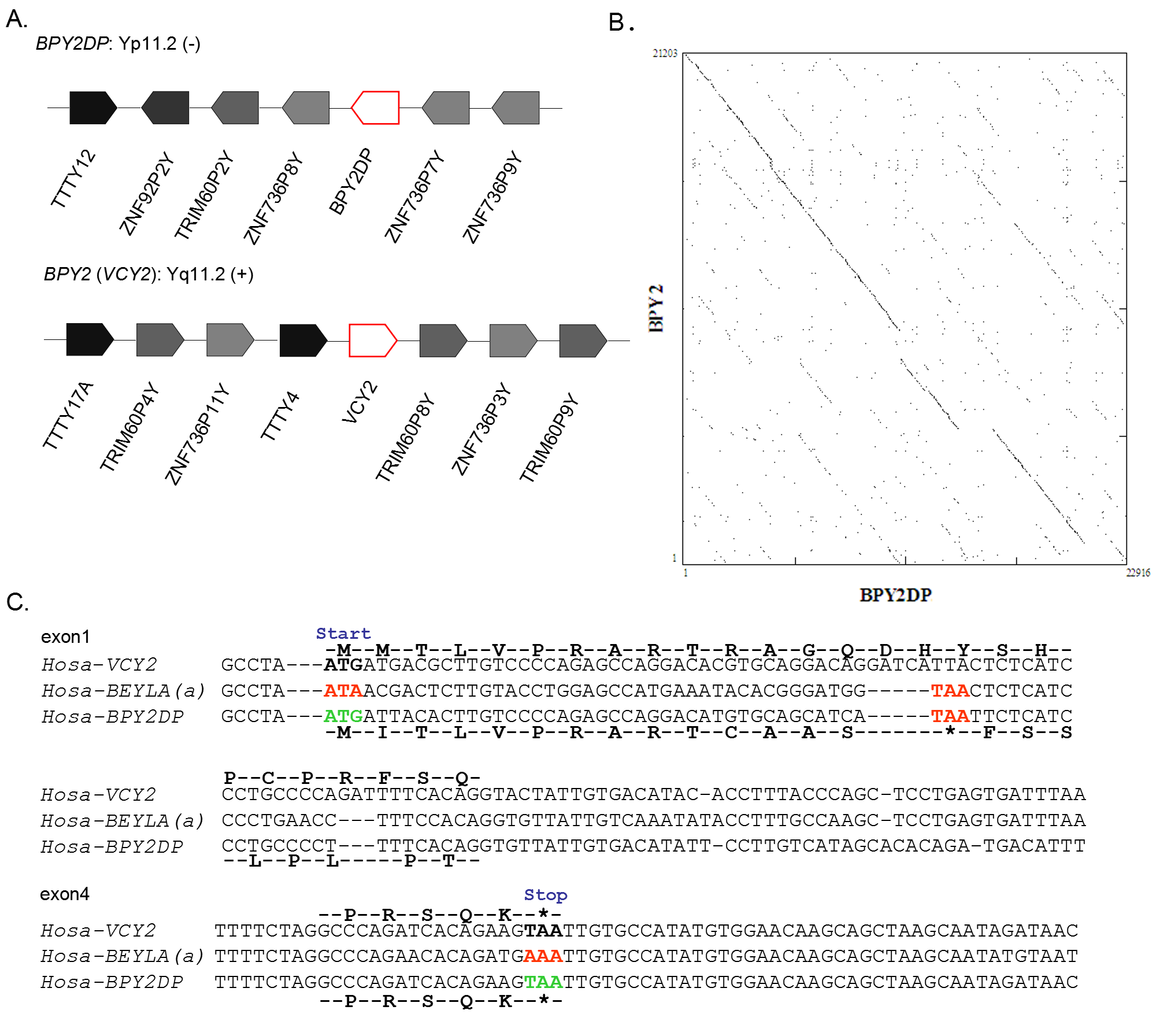

Supplement: S7 Fig — The synteny block comparison around human pseudogene gene BPY2DP and VCY2 (BPY2). The order and orientation for each gene in the green-amplicon was shown. (B) The dot-plot analysis for BPY2DP and VCY2 genomic sequences. Each dot in the map represent a fragment of 14 bp length (mismatch is 0) in the alignment. (C). The alignment of the BPY2DP and partial protein-coding sequences of VCY2, the deduced protein sequence for VCY2 exon 1 and exon 4 above the row. Positions of start codons are indicated. Disablers are indicated by red. Red and green arrow indicate the enabler and disabler for BPY2DP. Hosa, Homo sapiens (TIF) [file pone.0119651.s007.tif]

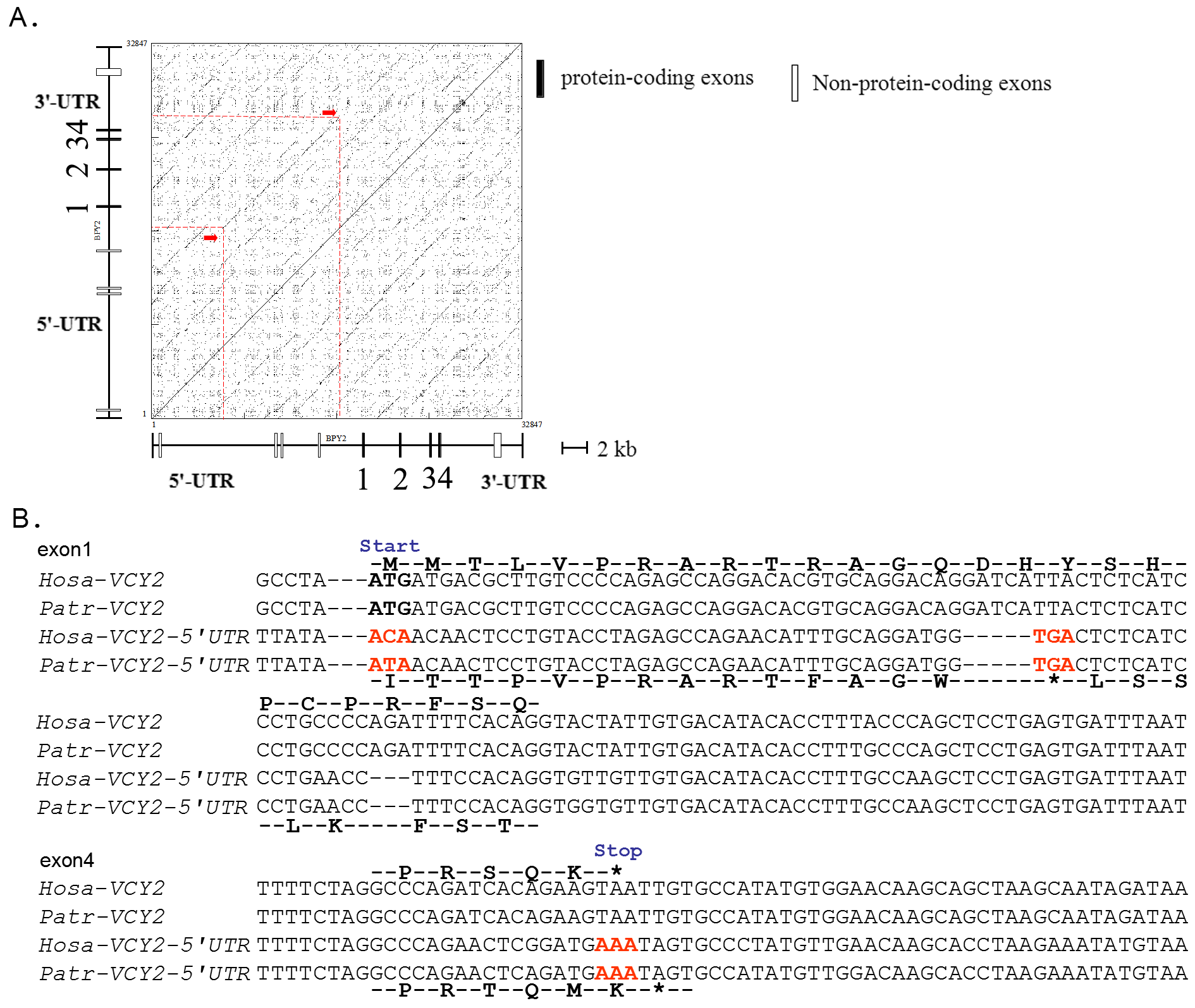

Supplement: S8 Fig — The dot-plot analysis of the human VCY2 genomic sequence with itself. Each dot in the map represent a fragment of 11 bp length (mismatch is 0) in the alignment. The exon-intron structure of human VCY2 was shown. The position of protein-coding exons of VCY2 were indicated as 1–4. The protein-coding exons (black bars) and non-protein-coding exons in 5’-UTR and 3’UTR (white bars); 1 cm in the diagram = 2 kb in the genomic sequence. The arrow and the red dash lines indicated the positions of the duplicated segments (B) Artificial translations of-VCY2 5’UTR ORF are shown below each row of the alignment and compared to the deduced protein sequence for VCY2 ORF above the row. Positions of start and stop codons are indicated. Disablers are indicated by red. Hosa, Homo sapiens; Patr, Pan troglodytes; UTR, untranslated region. (TIF) [file pone.0119651.s008.tif]
